# Central Sensitization and Pain Catastrophizing Are More Strongly Associated with Health-Related Quality of Life than Bioelectrical Impedance Analysis-Derived Body-Composition Measures in Patients with Chronic Low Back Pain

**DOI:** 10.3390/diagnostics16182913

**Published:** 2026-09-09

**Authors:** Sebastian Tirla, Carmen Delia Nistor-Cseppento, Brigitte Osser, Dorina Maria Farcas, Nicoleta Anamaria Pascalau, Cristian Marge, Gyongyi Osser, Laura-Ioana Radoi

**Affiliations:** 1 Doctoral School of Biomedical Sciences, Faculty of Medicine and Pharmacy, University of Oradea, 410073 Oradea, Romania; tirla.sebastian@student.uoradea.ro (S.T.);; 2 Department of Psycho Neuroscience and Recovery, Faculty of Medicine and Pharmacy, University of Oradea, 410073 Oradea, Romanianicoleta.pascalau@didactic.uoradea.ro (N.A.P.);; 3 Faculty of Physical Education and Sport, “Aurel Vlaicu” University of Arad, 310130 Arad, Romania; 4 Master’s Programme in Management of Musculoskeletal Disorders, Faculty of Medicine and Pharmacy, University of Oradea, 410087 Oradea, Romania; 5 Department of Biology and Life Sciences, Faculty of Medicine, “Vasile Goldiș” Western University of Arad, 310048 Arad, Romania; bondar.laura@uvvg.ro

**Keywords:** bioelectrical impedance analysis, central sensitization, chronic pain, health-related quality of life, low back pain, pain catastrophizing, rehabilitation

## Abstract

**Background/Objectives**: Chronic low back pain (LBP) is a multifactorial condition in which biological, psychological, and physical factors are associated with pain persistence and reduced health-related quality of life (HRQoL). Although central sensitization, pain catastrophizing, functional disability, and body composition have each been associated with CLBP, their relative contributions to HRQoL remain incompletely understood. This study investigated the relationships among these factors and identified factors associated with physical and mental HRQoL in multivariable analyses. **Methods**: In this cross-sectional observational study, 97 adults with CLBP undergoing rehabilitation were consecutively recruited from a tertiary rehabilitation department. Participants underwent standardized clinical evaluation, psychometric assessment using the Central Sensitization Inventory-9 (CSI-9), Pain Catastrophizing Scale (PCS), Roland–Morris Disability Questionnaire (RMDQ), and Italian Chronic Pain Classification (ICDS), whole-body bioelectrical impedance analysis (BIA), and HRQoL assessment using the 36-Item Short Form Health Survey (SF-36). Correlation analyses, group comparisons according to pain chronicity, and multivariable linear regression analyses were performed to evaluate factors associated with the SF-36 Physical Component Summary (SF-36 PCS) and SF-36 Mental Component Summary (SF-36 MCS) scores after adjustment for the variables included in the models. **Results**: Greater central sensitization, pain catastrophizing, and functional disability were associated with poorer HRQoL, whereas the evaluated BIA-derived body-composition measures showed weak or non-significant associations. Patients with higher degrees of pain chronicity demonstrated significantly higher central sensitization, pain catastrophizing, and disability, together with lower physical and mental HRQoL, but did not differ significantly in body composition. In multivariable analyses, central sensitization and pain catastrophizing remained associated with both SF-36 PCS and SF-36 MCS scores after adjustment for the other variables included in the models, whereas body composition parameters were not significantly associated with HRQoL. **Conclusions**: Psychological pain-related factors, particularly central sensitization and pain catastrophizing, are more strongly associated with HRQoL than the evaluated BIA-derived body-composition measures in patients with CLBP. Assessment of these factors may help inform patient stratification and personalized biopsychosocial rehabilitation planning; however, whether interventions specifically targeting them improve HRQoL requires prospective evaluation.

## 1. Introduction

Chronic low back pain (LBP) is one of the leading causes of disability worldwide and represents a major public health challenge, affecting quality of life, work productivity, and healthcare utilization. Although most individuals experience LBP during their lifetime, approximately 5–10% develop persistent symptoms lasting longer than three months, resulting in substantial personal, social, and economic burden [1,2,3]. Increasing evidence indicates that CLBP is not solely a structural or biomechanical disorder but rather a complex biopsychosocial condition in which biological, psychological, and social factors interact and are associated with pain persistence, disability, and treatment outcomes [4,5,6].

Among the biological mechanisms implicated in chronic pain, central sensitization has emerged as an important factor associated with persistent pain and functional impairment. It is characterized by increased responsiveness of central nociceptive pathways, leading to pain hypersensitivity that cannot be fully explained by peripheral tissue pathology alone [7,8,9]. Patients with greater central sensitization frequently report higher pain intensity, greater disability, reduced physical functioning (PF), and poorer health-related quality of life (HRQoL) than patients without evidence of central sensitization [10,11,12]. Consequently, the assessment of central sensitization has become an increasingly important component of the clinical evaluation of patients with CLBP.

Psychological factors are equally important in the development and maintenance of chronic pain. Pain catastrophizing, characterized by rumination, magnification of pain-related thoughts, and feelings of helplessness, has consistently been associated with increased pain severity, disability, emotional distress, and poorer rehabilitation outcomes [4,6,13]. Furthermore, catastrophizing is believed to interact with central sensitization through maladaptive pain processing, potentially amplifying symptom severity and limiting functional recovery [14,15,16]. Together, these findings support the concept that biological and psychological mechanisms should be evaluated simultaneously when assessing patients with chronic LBP.

HRQoL has become one of the most relevant patient-reported outcomes in chronic pain research because it reflects the multidimensional impact of persistent pain on physical, psychological, and social functioning (SF). Although impaired HRQoL is consistently observed in patients with CLBP, the factors associated with this impairment remain incompletely understood. Previous studies have reported associations between reduced HRQoL and central sensitization, pain catastrophizing, functional disability, and pain chronicity; however, the relative contribution of these factors remains uncertain [17,18,19].

Body composition has also been proposed as a potential factor associated with chronic LBP. Obesity and excess adiposity have been associated with increased mechanical loading, low-grade systemic inflammation, and metabolic dysregulation, whereas reduced skeletal muscle mass and sarcopenia may impair spinal stability and physical performance [20,21,22]. Nevertheless, published findings remain inconsistent, and it is still unclear whether body composition is independently associated with reduced HRQoL after accounting for psychological and clinical characteristics [2,23].

Despite the growing body of evidence regarding individual factors associated with chronic LBP, few studies have simultaneously evaluated central sensitization, pain catastrophizing, functional disability, pain chronicity, body composition, and HRQoL within the same cohort of patients undergoing rehabilitation. Furthermore, the relative contribution of psychological factors and body composition to both physical and mental components of HRQoL remains insufficiently understood. Addressing this knowledge gap may improve patient phenotyping, facilitate individualized rehabilitation strategies, and help identify modifiable factors associated with impaired HRQoL.

We hypothesized that psychological pain-related factors would show stronger associations with HRQoL than body composition parameters in patients with chronic LBP. Therefore, the aim of this study was to evaluate the relationships among central sensitization, pain catastrophizing, functional disability, body composition, pain chronicity, and HRQoL in patients with chronic LBP undergoing rehabilitation, and to identify factors associated with physical and mental HRQoL in multivariable regression analysis.

## 2. Materials and Methods

### 2.1. Study Design and Participants

This cross-sectional observational study was conducted to investigate the relationships among central sensitization, pain catastrophizing, body composition, pain chronicity, functional disability, and HRQoL in adults with chronic LBP.

Participants were consecutively recruited from the Department of Physical Medicine and Rehabilitation of the Clinical Emergency Hospital “Avram Iancu”, Oradea, Romania, between January and December 2025. During a single study visit, all participants underwent a standardized clinical evaluation, anthropometric measurements, body composition assessment, psychometric evaluation, and HRQoL assessment.

Eligible participants met all of the following inclusion criteria:

- Age ≥ 18 years;
- Clinical diagnosis of chronic LBP, defined as pain localized between the lower costal margin and the inferior gluteal folds, with or without referred pain to the lower extremities, persisting for at least 12 weeks;
- Diagnosis established by a consultant in Physical Medicine and Rehabilitation based on clinical history, physical examination, and review of imaging findings when clinically indicated.

Participants were excluded if they met any of the following criteria:

- Specific spinal pathology (including vertebral fracture, spinal infection, inflammatory spinal disease, spinal malignancy, or cauda equina syndrome);
- Serious neurological disorders affecting mobility;
- Pregnancy;
- Implanted electronic medical devices contraindicating bioelectrical impedance analysis (BIA);
- Severe cognitive impairment precluding reliable completion of the questionnaires.

**Figure 1:**
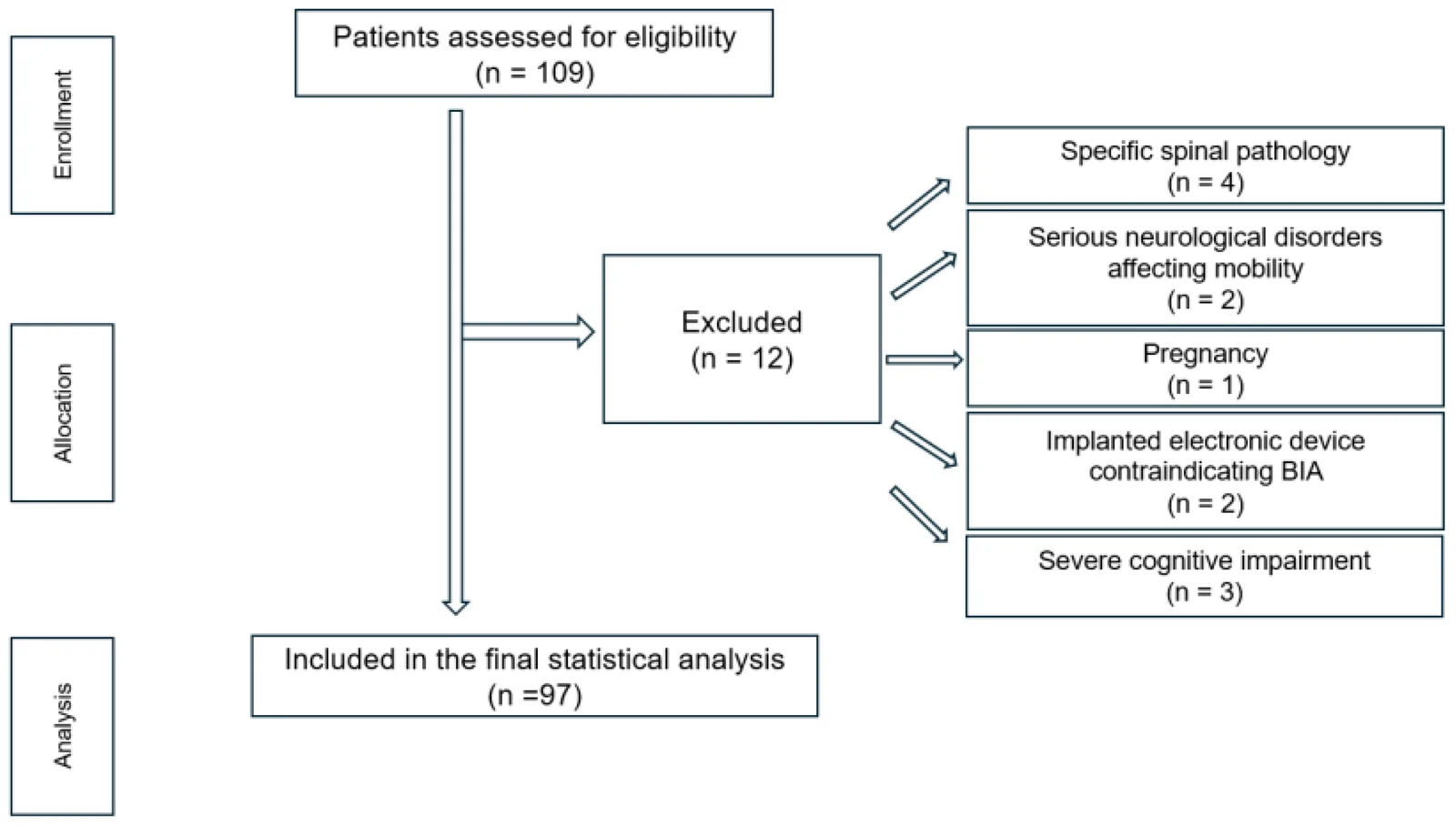
Flow diagram illustrating participant screening, eligibility assessment, exclusions, and inclusion in the final statistical analysis.

A total of 97 participants fulfilled the eligibility criteria, completed all study assessments, and were included in the final statistical analyses. The participant recruitment and selection process is illustrated in Figure 1.

### 2.2. Clinical Assessment

Clinical assessment was performed by a consultant in Physical Medicine and Rehabilitation using a standardized evaluation protocol. Demographic characteristics, including age, sex, and place of residence, were recorded. Anthropometric measurements included body weight and height, from which body mass index (BMI) was calculated as weight (kg) divided by height squared (m^2^). Participants were classified as having normal weight, being overweight, or having obesity according to the World Health Organization BMI classification.

Clinical characteristics included the underlying diagnosis of chronic LBP (vertebrogenic, discogenic, mixed, or lumbar spinal stenosis) established through clinical examination and imaging findings when clinically indicated. Comorbidity burden was assessed using the Charlson Comorbidity Index (CCI), a validated instrument that estimates the cumulative impact of chronic diseases on overall health status. Diabetes status was additionally recorded from the participants’ medical records and was considered in an exploratory analysis evaluating potential effect modification of the association between visceral adiposity and HRQoL.

Routine laboratory parameters, including erythrocyte sedimentation rate (ESR), total cholesterol, and triglyceride concentrations, were obtained from medical records and were used to characterize the study population.

Habitual physical activity, previous musculoskeletal events or conditions (e.g., fragility fractures, major lower-extremity fractures, arthroplasty, or previous spinal surgery), and actual pain duration beyond the ≥12-week inclusion criterion were not systematically collected and were therefore not included in the present analyses.

Following the clinical evaluation, participants completed standardized psychometric questionnaires, underwent HRQoL assessment, and received whole-body BIA as described below.

### 2.3. Psychometric Assessment

Psychometric assessment was performed using self-administered questionnaires completed by all participants during the study visit. The evaluated domains included central sensitization, pain catastrophizing, functional disability, and pain chronicity.

Central sensitization was assessed using the 9-item Central Sensitization Inventory (CSI-9), a validated short-form version of the original Central Sensitization Inventory. The questionnaire comprises nine items scored on a 5-point Likert scale (0–4), yielding a total score ranging from 0 to 36, with higher scores indicating greater symptoms associated with central sensitization.

Pain catastrophizing was evaluated using the Pain Catastrophizing Scale (PCS). The PCS consists of 13 items assessing three domains of pain-related cognition: rumination, magnification, and helplessness. Each item is scored on a 5-point Likert scale (0–4), resulting in a total score ranging from 0 to 52, with higher scores reflecting greater levels of catastrophic thinking related to pain.

Functional disability was assessed using the Roland–Morris Disability Questionnaire (RMDQ), one of the most widely used disease-specific instruments for evaluating disability associated with chronic LBP. The questionnaire contains 24 dichotomous (yes/no) items, with total scores ranging from 0 to 24. Higher scores indicate greater disability.

Pain chronicity was evaluated using the Italian Chronic Pain Classification (ICDS), which assesses the degree of pain chronicity based on multiple clinical dimensions of chronic pain. Total scores range from 0 to 14, with higher scores indicating a greater degree of pain chronicity. For subgroup analyses, participants were categorized as having a lower degree of pain chronicity (ICDS < 8) or higher degree of pain chronicity (ICDS ≥ 8). However, the psychometric properties and cross-cultural validity of the ICDS remain insufficiently documented outside Italian-speaking populations, and the instrument has not been specifically validated in a Romanian population. Accordingly, ICDS-based subgroup findings were interpreted cautiously.

### 2.4. HRQoL Assessment

HRQoL was assessed using the 36-Item Short Form Health Survey (SF-36), a widely validated generic instrument for evaluating perceived health status across multiple dimensions of physical and mental well-being. The questionnaire consists of 36 items grouped into eight domains: PF, Role Limitations due to Physical Health (RP), Bodily Pain (BP), General Health (GH), Vitality (VT), SF, Role Limitations due to Emotional Problems (RE), and Mental Health (MH).

Raw domain scores were transformed to a standardized 0–100 scale according to the SF-36 scoring manual, with higher scores indicating better perceived health status and quality of life. In addition to the eight domain scores, two summary measures were calculated: the SF-36 Physical Component Summary (SF-36 PCS) and the SF-36 Mental Component Summary (SF-36 MCS), representing overall physical and MH status, respectively.

The SF-36 domain scores and summary component scores were used as the primary outcome measures in the statistical analyses to investigate their associations with central sensitization, pain catastrophizing, body composition, pain chronicity, and functional disability.

### 2.5. Body Composition Assessment

Body composition was assessed using a Tanita MC-780 Multi Frequency Segmental Body Composition Analyzer (Tanita Corporation, Tokyo, Japan), which estimates body composition through multifrequency BIA. Measurements were performed by trained personnel according to the manufacturer’s standardized protocol.

Participants were instructed to avoid vigorous physical activity, alcohol consumption, and large meals prior to the assessment and to remove shoes, socks, and heavy clothing before measurement. Body weight was measured to the nearest 0.1 kg, while standing height was measured using a calibrated stadiometer to the nearest 0.1 cm. BMI was calculated as weight (kg) divided by height squared (m^2^).

The BIA assessment generated whole-body and segmental estimates of body composition, including body fat percentage (%), fat mass (kg), fat-free mass (kg), skeletal muscle mass (kg), total body water (%), visceral fat rating, and basal metabolic rate (kcal/day). For the present analyses, skeletal muscle index (SMI) was calculated as whole-body skeletal muscle mass (kg) divided by height squared (m^2^). Appendicular skeletal muscle mass was not calculated or used in the present analyses; therefore, the reported SMI represents a whole-body skeletal muscle mass index and should not be interpreted as appendicular skeletal muscle mass index (ASMI). No sarcopenia classification was applied because muscle strength and physical performance were not specifically assessed for this purpose.

### 2.6. Statistical Analysis

Statistical analyses were performed using IBM SPSS Statistics software (Version 29.0; IBM Corp., Armonk, NY, USA). Statistical significance was established at a two-sided *p*-value < 0.05.

Continuous variables were assessed for normality using the Shapiro–Wilk test and by visual inspection of histograms and Q–Q plots. Normally distributed variables are presented as mean ± standard deviation (SD), whereas non-normally distributed variables are presented as median and interquartile range (IQR). Categorical variables are expressed as absolute frequencies and percentages.

Comparisons between participants with low and higher degrees of pain chronicity were performed using the independent-samples *t*-test or the Mann–Whitney U test, as appropriate, for continuous variables, and the chi-square test or Fisher’s exact test for categorical variables. Associations among central sensitization, pain catastrophizing, functional disability, body composition parameters, pain chronicity, and HRQoL were evaluated using Pearson’s correlation coefficient. Because some variables, particularly the ICDS score, showed non-normal distributions, Spearman’s rank correlation coefficients were additionally calculated as a sensitivity analysis to assess the robustness of the correlation findings to distributional assumptions.

To identify factors associated with HRQoL, separate multivariable linear regression models were constructed using the SF-36 PCS and SF-36 MCS scores as dependent variables. Candidate independent variables were selected based on clinical relevance, previous literature, and their associations with HRQoL in the correlation analyses. The final models included CSI-9 score, PCS total score, RMDQ score, body fat percentage, and SMI. All selected independent variables were entered simultaneously into each model using the standard enter method.

Age and sex were not included in the primary models in order to preserve model parsimony given the sample size and the prespecified focus on psychological, functional, and body-composition factors; however, because both variables are clinically relevant potential confounders, their influence was examined in sensitivity analyses.

To evaluate the robustness of the primary findings to potential demographic confounding, sensitivity analyses were performed by additionally including age and sex in both multivariable models. Age and sex were included irrespective of their associations in the correlation analyses because of their potential relationships with body composition, functional status, and HRQoL. The same modelling strategy and diagnostic procedures were applied to the sensitivity models.

An additional sensitivity analysis was performed to evaluate visceral adiposity as an alternative measure of adiposity. In these models, visceral fat level replaced body fat percentage, while CSI-9 score, PCS total score, RMDQ score, SMI, age, and sex were retained as covariates. Separate models were constructed for the SF-36 PCS and SF-36 MCS outcomes using the same enter method and model diagnostic procedures. Body fat percentage and visceral fat level were not included simultaneously because of their conceptual overlap as measures of adiposity and their correlation within the study cohort.

Model assumptions were evaluated by assessing linearity, normality and homoscedasticity of residuals, independence of errors, and multicollinearity. Multicollinearity was assessed using tolerance and variance inflation factor (VIF) values, with VIF values < 5 considered indicative of the absence of problematic multicollinearity.

Sample size adequacy was evaluated using G*Power software (version 3.1.9.7; Heinrich Heine University Düsseldorf, Düsseldorf, Germany) for the primary multivariable linear regression analyses. Assuming a medium effect size (Cohen’s f^2^ = 0.15), five predictors, a significance level of α = 0.05, and 80% statistical power, the estimated minimum sample size was 92 participants. The final sample of 97 participants therefore met the estimated sample size requirement.

### 2.7. Ethical Considerations

The study was conducted in accordance with the ethical principles of the Declaration of Helsinki and its subsequent amendments. The study protocol was reviewed and approved by the Ethics Committee of the Clinical Emergency Hospital “Avram Iancu”, Oradea, Romania (Approval No. 1118263/30 May 2024). Written informed consent was obtained from all participants prior to enrollment, and all data were anonymized before statistical analysis to ensure participant confidentiality.

## 3. Results

### 3.1. Cohort Characteristics

A total of 97 patients completed all study assessments and were included in the final analysis. The study population was predominantly female (72/97, 74.2%), with a mean age of 66.6 ± 9.2 years. Participants ranged in age from 45 to 84 years, with a median age of 68 years (IQR, 61–73). Obesity was highly prevalent, with a mean BMI of 33.1 ± 6.1 kg/m^2^, and 68 participants (70.1%) were classified as obese. The median CCI was 3.0 (IQR, 2.0–5.0), indicating a moderate burden of chronic comorbidities. Diabetes was present in 25 participants (25.8% of the study population).

Vertebrogenic LBP was the most frequent clinical diagnosis (67.0%), followed by mixed (19.6%) and discogenic (12.4%) pain. Overall, the study population was characterized by older age, a high prevalence of obesity, and multiple chronic comorbidities. Detailed demographic, anthropometric, clinical, and laboratory characteristics are presented in Table 1.

### 3.2. Psychometric Characteristics, Disability, and HRQoL

Patients demonstrated moderate levels of central sensitization and pain catastrophizing, accompanied by moderate functional disability and impaired HRQoL. The mean CSI-9 score was 18.9 ± 7.4, while the mean PCS total score was 22.8 ± 13.8. Functional disability, assessed using the RMDQ, averaged 10.1 ± 6.2 points. More than half of the study population (56/97, 57.7%) met the predefined threshold for higher degree of pain chronicity (ICDS ≥ 8).

HRQoL was predominantly impaired in the physical domain. The mean SF-36 PCS score was 35.5 ± 17.0, whereas the SF-36 MCS score was comparatively higher (49.6 ± 15.2), indicating greater impairment in physical than MH. Among the individual SF-36 domains, RP, BP, and GH demonstrated the lowest scores, whereas MH showed the highest average score. Detailed psychometric, disability, pain chronicity, and health-related quality-of-life outcomes are presented in Table 2.

### 3.3. Body Composition Profile

Whole-body BIA revealed a body-composition profile characterized by increased adiposity. The mean body fat percentage was 31.8 ± 10.9%, while the mean visceral fat level was 11.9 ± 5.1. Mean whole-body skeletal muscle mass was 30.4 ± 5.7 kg, corresponding to a mean whole-body SMI of 8.5 ± 1.3 kg/m^2^.

Because the SMI used in the present study was derived from whole-body skeletal muscle mass rather than appendicular skeletal muscle mass, and muscle strength and physical performance were not assessed for the purpose of diagnosing sarcopenia, participants were not classified according to sarcopenia status. Detailed body-composition parameters are presented in Table 3.

### 3.4. Comparison According to Pain Chronicity

Patients were stratified according to pain chronicity using the ICDS. Fifty-six participants (57.7%) were classified as having higher degrees of pain chronicity (ICDS ≥ 8), whereas 41 (42.3%) had lower degrees of pain chronicity (ICDS < 8).

Patients with higher degrees of pain chronicity exhibited a markedly greater psychosocial burden than those with lower degrees of pain chronicity. Specifically, they demonstrated significantly higher levels of central sensitization, pain catastrophizing, and functional disability, accompanied by substantially poorer physical and mental HRQoL. In contrast, demographic characteristics, anthropometric measures, body composition, and laboratory parameters did not differ significantly between groups. Detailed between-group comparisons are presented in Table 4.

### 3.5. Correlation Analysis

Correlation analysis revealed strong interrelationships among central sensitization, pain catastrophizing, functional disability, pain chronicity, and HRQoL. Higher CSI-9 scores were strongly correlated with higher PCS total scores and greater functional disability, while showing moderate-to-strong inverse correlations with both the SF-36 PCS and SF-36 MCS scores.

Pain catastrophizing was also positively correlated with functional disability and pain chronicity and negatively correlated with HRQoL, indicating that greater psychological distress was associated with poorer physical and mental functioning. In contrast, body composition variables, including body fat percentage, visceral fat level, and SMI, showed weak or non-significant correlations with SF-36 outcomes.

**Figure 2:**
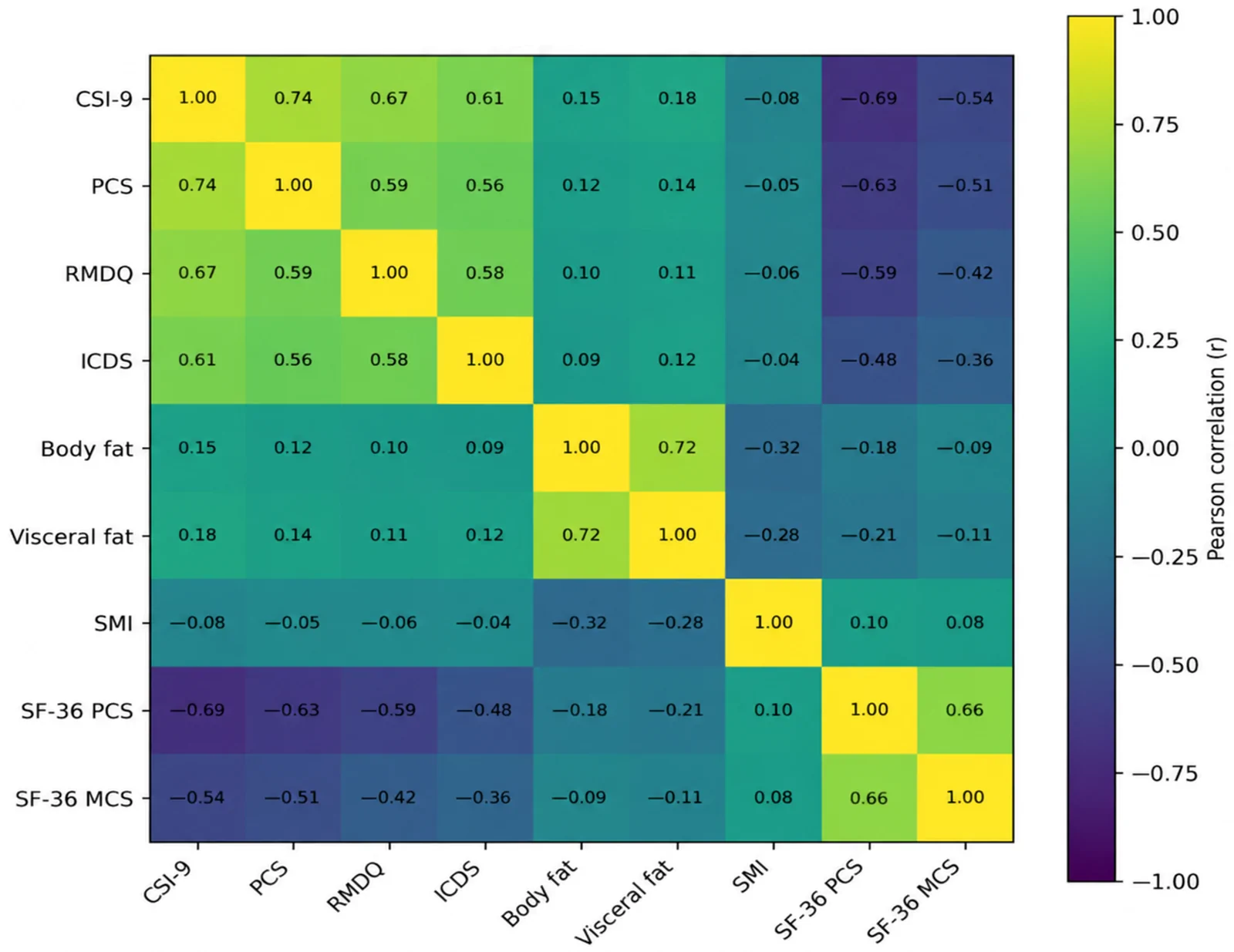
Pearson correlation matrix of psychometric characteristics, disability, body composition, pain chronicity, and HRQoL.

**Figure 3:**
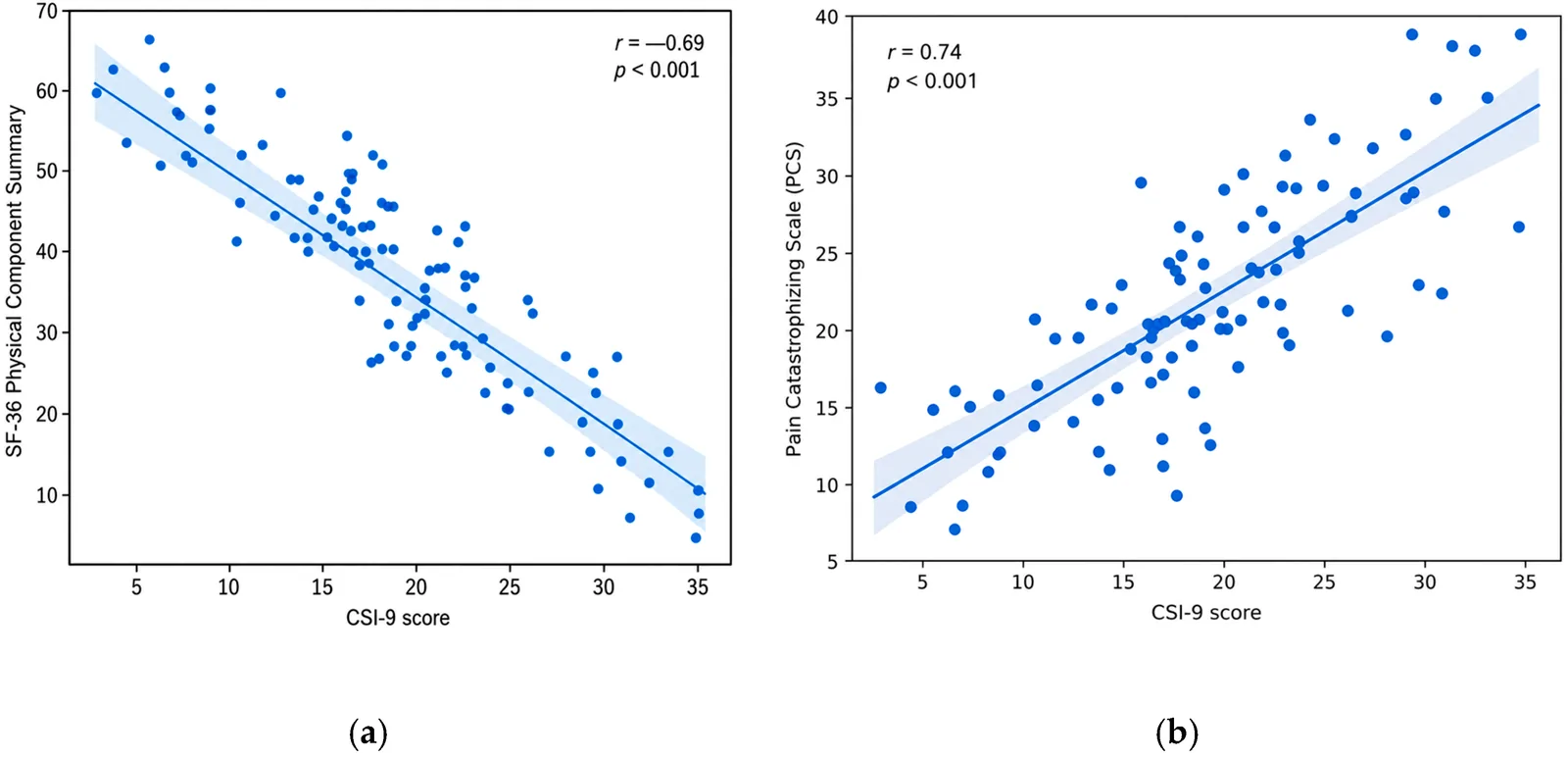
Scatterplots showing the associations between CSI-9 score and (**a**) SF-36 PCS and (**b**) PCS total score.

Overall, within the variables evaluated in this cohort, central sensitization and pain catastrophizing exhibited the strongest statistical associations with HRQoL. These findings, together with clinical relevance and evidence from previous literature, informed the subsequent multivariable regression analyses. A comprehensive correlation matrix is presented in Figure 2, whereas the strongest clinically relevant associations are illustrated in Figure 3.

Because some variables, particularly ICDS, showed non-normal distributions, Spearman’s rank correlations were additionally calculated as a sensitivity analysis. The Spearman analysis yielded a pattern of associations broadly consistent with the primary Pearson analysis. In particular, ICDS remained positively associated with central sensitization, pain catastrophizing, and functional disability and inversely associated with physical and mental HRQoL. These findings supported the robustness of the main correlation findings to the choice of correlation method (Table 5).

### 3.6. Multivariable Factors Associated with HRQoL

Multivariable linear regression analyses were performed separately for the SF-36 PCS and SF-36 MCS scores. Inspection of the residual diagnostics indicated no substantial violations of linearity, normality, or homoscedasticity, and the independence-of-errors assumption was adequately met. No evidence of problematic multicollinearity was observed among the included predictors, with VIF values ranging from 1.5 to 2.4, all below the predefined threshold of 5.

For the SF-36 PCS, higher CSI-9 scores, higher pain catastrophizing scores, and greater functional disability (RMDQ) were associated with lower SF-36 PCS scores after adjustment for the other variables included in the model. Body fat percentage and SMI were not significantly associated with SF-36 PCS in the model.

**Figure 4:**
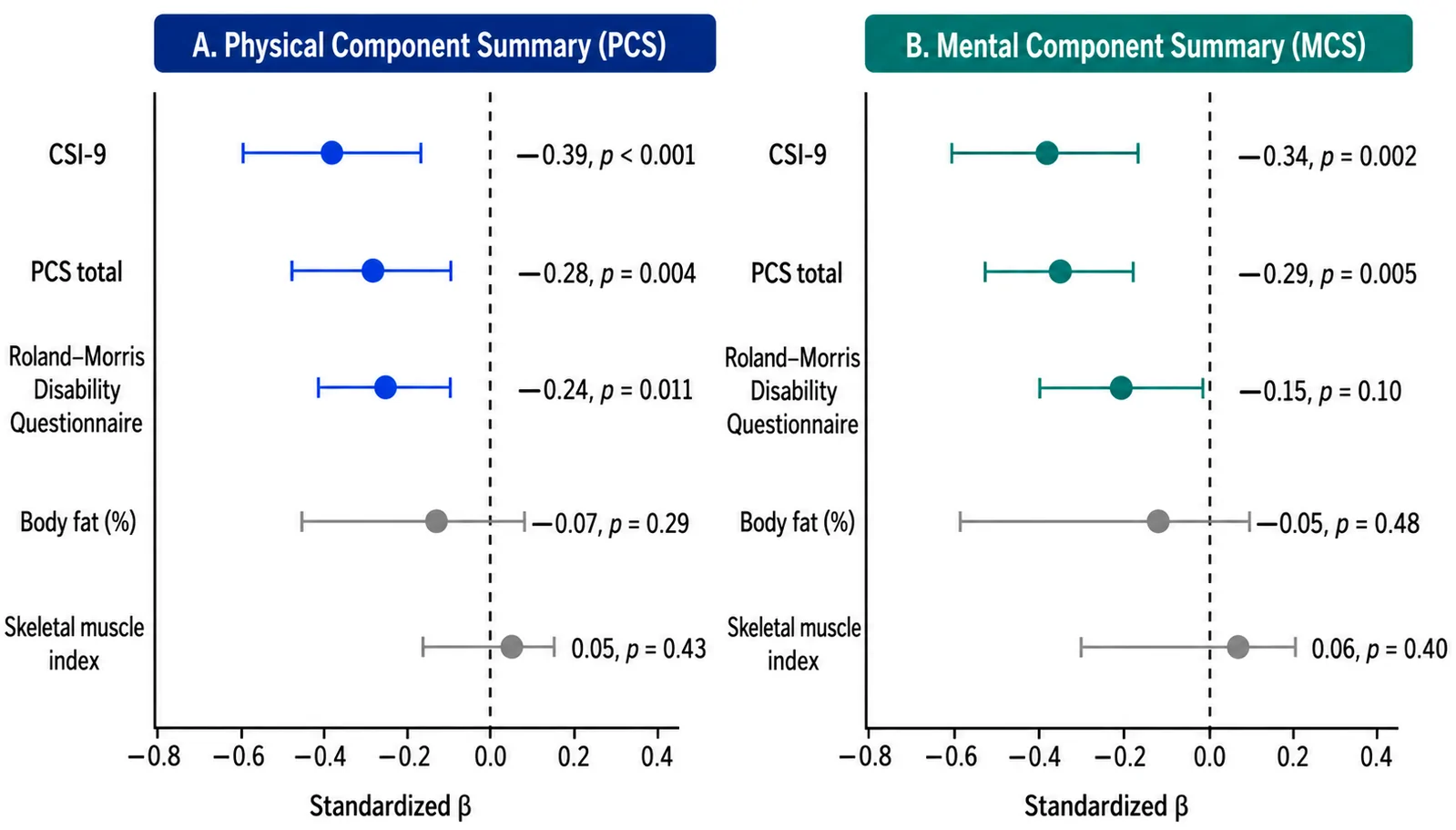
Forest plot of standardized regression coefficients from multivariable linear regression models examining multivariable associations with physical (**A**) and mental (**B**) HRQoL. Blue and green distinguish the physical and mental HRQoL models, respectively; gray indicates body-composition variables.

For the SF-36 MCS, higher CSI-9 and pain catastrophizing scores were associated with lower SF-36 MCS scores after adjustment for the other variables included in the model, whereas functional disability showed a weaker and non-significant association. Body fat percentage and SMI were not significantly associated with SF-36 MCS. Detailed regression coefficients and multicollinearity diagnostics are presented in Table 6, whereas standardized effect sizes are illustrated in Figure 4.

### 3.7. Sensitivity Analyses Adjusted for Age and Sex

Sensitivity analyses were performed to evaluate whether the associations observed in the primary models remained evident after additional adjustment for age and sex. In the age- and sex-adjusted SF-36 PCS model, higher CSI-9, pain catastrophizing, and RMDQ scores remained significantly associated with poorer physical HRQoL, whereas body fat percentage, SMI, age, and sex were not significantly associated with SF-36 PCS. The overall SF-36 PCS model was statistically significant (adjusted R^2^ = 0.60, *p* < 0.001).

Similarly, in the age- and sex-adjusted SF-36 MCS model, higher CSI-9 and pain catastrophizing scores remained significantly associated with poorer mental HRQoL, whereas RMDQ, body fat percentage, SMI, age, and sex were not significantly associated with SF-36 MCS. The overall SF-36 MCS model was statistically significant (adjusted R^2^ = 0.48, *p* < 0.001). No problematic multicollinearity was identified, with VIF values ranging from 1.64 to 2.45. Detailed results of the sensitivity analyses are presented in Table 7.

### 3.8. Sensitivity Analyses Incorporating Visceral Fat

Additional sensitivity analyses were performed to examine whether visceral adiposity was associated with HRQoL when considered as an alternative adiposity measure. In these models, visceral fat level replaced body fat percentage, while CSI-9, pain catastrophizing, RMDQ, SMI, age, and sex were retained as covariates.

In the SF-36 PCS model, higher CSI-9, pain catastrophizing, and RMDQ scores remained significantly associated with poorer physical HRQoL, whereas visceral fat level, SMI, age, and sex were not significantly associated with SF-36 PCS. The overall model was statistically significant (adjusted R^2^ = 0.59, *p* < 0.001).

Similarly, in the SF-36 MCS model, higher CSI-9 and pain catastrophizing scores remained significantly associated with poorer mental HRQoL, whereas visceral fat level, RMDQ, SMI, age, and sex were not significantly associated with SF-36 MCS. The overall model was statistically significant (adjusted R^2^ = 0.47, *p* < 0.001). No problematic multicollinearity was identified, with all VIF values below 5. Detailed results are presented in Table 8.

In an additional exploratory analysis, the interaction between visceral fat level and diabetes status was not statistically significant for either physical HRQoL (SF-36 PCS: B = −0.07, 95% CI −0.34 to 0.20, *p* = 0.61) or mental HRQoL (SF-36 MCS: B = −0.09, 95% CI −0.37 to 0.19, *p* = 0.52). These findings did not provide evidence that diabetes modified the association between visceral adiposity and HRQoL in this cohort (Table 9).

## 4. Discussion

The present study investigated the relationships among central sensitization, pain catastrophizing, functional disability, body composition, pain chronicity, and HRQoL in patients with chronic LBP undergoing rehabilitation. Central sensitization and pain catastrophizing showed the strongest multivariable associations with physical and mental HRQoL. Importantly, these associations remained evident in sensitivity analyses additionally adjusting for age and sex, suggesting that the principal findings were robust to adjustment for these demographic characteristics. Functional disability remained significantly associated with physical HRQoL after adjustment for the other variables included in the model, whereas body fat percentage and SMI were not significantly associated with either HRQoL component.

Participants with higher pain chronicity also exhibited greater central sensitization, pain catastrophizing, and functional disability, together with poorer physical and mental HRQoL, while anthropometric and body composition parameters did not differ significantly between pain chronicity groups. Overall, these findings are consistent with a biopsychosocial interpretation of chronic LBP and indicate that central pain-processing, psychological, and functional characteristics showed stronger statistical associations with HRQoL than the evaluated body composition parameters within this rehabilitation cohort [24,25].

### 4.1. Central Sensitization and Pain Catastrophizing

The prominent associations of central sensitization and pain catastrophizing with HRQoL support the study hypothesis and are consistent with a biopsychosocial understanding of chronic LBP [9,26,27].

Higher levels of central sensitization have previously been associated with greater pain severity, disability, poorer PF, and reduced quality of life in patients with chronic musculoskeletal pain [11,28,29]. Central sensitization involves enhanced responsiveness of central nociceptive pathways, which may amplify pain perception and contribute to persistent symptoms that are not fully explained by peripheral tissue pathology [30,31,32]. This altered pain processing may therefore be associated with the functional limitations and poorer rehabilitation outcomes reported in patients with greater sensitization [33,34,35,36].

Pain catastrophizing was similarly associated with greater disability, more advanced pain chronicity, and poorer physical and mental HRQoL and remained significantly associated with both HRQoL components after adjustment for the other variables included in the models. This is consistent with previous evidence linking catastrophizing with disability, pain persistence, and reduced quality of life in chronic pain populations [13,37,38]. Increased attention to pain, maladaptive avoidance, emotional distress, and ineffective coping represent potential mechanisms underlying the association between catastrophic thinking and greater symptom and functional burden [15,25,39].

Central sensitization and pain catastrophizing were also closely correlated, suggesting an interaction between central pain processing and cognitive–emotional responses. Previous evidence indicates that catastrophic cognitions may be associated with enhanced pain processing through hypervigilance and emotional distress, while persistent pain hypersensitivity may reinforce negative pain-related beliefs [14,24,40]. However, given the cross-sectional design of the present study, the direction of this relationship cannot be established. Their associations with HRQoL after multivariable adjustment nevertheless suggest that these constructs capture related but distinct dimensions of the chronic pain experience.

Finally, functional disability remained significantly associated with physical HRQoL after adjustment for the other variables included in the model but showed a weaker, non-significant association with mental HRQoL. This pattern suggests that disability is more closely related to the physical dimension of HRQoL, whereas central sensitization and pain catastrophizing were associated with both physical and mental components. Together, these findings are consistent with biopsychosocial models in which the burden of chronic LBP reflects the interaction of functional impairment, altered pain processing, and psychological factors [41,42,43,44,45,46].

### 4.2. Body Composition and HRQoL

Body composition has been increasingly recognized as a potential factor associated with chronic LBP, as obesity, excess adiposity, and reduced skeletal muscle mass may be related to pain through biomechanical and metabolic mechanisms [20,47,48]. In the present study, most participants were classified as overweight or obese. Nevertheless, body fat percentage, visceral fat level, and SMI showed only weak correlations with HRQoL, and body fat percentage and SMI were not significantly associated with either physical or mental HRQoL in the multivariable models. The small absolute correlation coefficients indicate that the evaluated body-composition parameters had only weak cross-sectional relationships with the SF-36 summary scores within this cohort. From a clinical perspective, this suggests that adiposity and skeletal muscle mass considered in isolation may have limited value for identifying differences in physical or mental HRQoL compared with the psychological and functional measures evaluated in this study.

These findings partially agree with previous studies reporting associations between obesity, greater pain intensity, reduced physical function, and poorer quality of life in patients with chronic LBP [21,49,50]. Potential mechanisms linking excess adiposity with chronic pain include increased mechanical loading, altered movement biomechanics, and low-grade systemic inflammation mediated by adipokines and pro-inflammatory cytokines [51,52,53].

Reduced skeletal muscle mass has likewise been associated with impaired physical performance and greater disability, particularly in older adults [54,55,56]. However, emerging evidence suggests that global body-composition measures may not fully capture clinically relevant regional differences. A recent DXA-based study in older women demonstrated that lower-limb muscle-mass asymmetry was associated with hip-fracture laterality, whereas global muscle indices showed less discriminatory value [57]. Similarly, a recent DXA study in older women with hip fracture found greater central adiposity in participants with type 2 diabetes despite no significant difference in appendicular lean mass index [58]. These findings emphasize that total adiposity, visceral or central adiposity, appendicular lean mass, and regional muscle characteristics represent related but distinct dimensions of body composition.

This distinction is relevant to the present cohort, which was predominantly older, female, and characterized by a high prevalence of obesity. Whole-body skeletal muscle mass was relatively preserved despite the high prevalence of obesity; however, the SMI used in this study was derived from whole-body skeletal muscle mass rather than appendicular or regional skeletal muscle mass. Therefore, potentially relevant regional differences or inter-limb asymmetry could not be evaluated, and the present findings should not be generalized to appendicular or regional muscle characteristics. Muscle strength was also not specifically assessed for sarcopenia diagnosis; consequently, the present findings should not be interpreted as indicating the presence or absence of sarcopenia according to contemporary consensus criteria.

Because visceral adiposity may also reflect metabolic risk, we additionally explored whether diabetes status modified the association between visceral fat and HRQoL. The visceral fat × diabetes interaction was not statistically significant for either the physical or mental component of the SF-36. Thus, within this cohort, we found no evidence that the association between visceral adiposity and HRQoL differed according to diabetes status. This exploratory finding should be interpreted cautiously, however, because the study was not primarily designed or powered to detect interaction effects, and subgroup sizes according to diabetes status may have limited statistical power. Larger studies specifically designed to investigate metabolic effect modification are warranted.

The weak associations observed in the present study are also consistent with evidence showing that relationships between obesity and quality of life may be attenuated after accounting for psychological distress, pain severity, and functional disability [17,53,59]. In the present study, body composition parameters were not statistically significant, whereas central sensitization and pain catastrophizing remained significantly associated with HRQoL after adjustment for the variables included in the models. Similarly, body composition did not differ significantly between participants with low and higher degree of pain chronicity, despite substantial differences in central sensitization, catastrophizing, disability, and HRQoL. However, these findings should be interpreted cautiously because the study population was markedly enriched for obesity, with approximately 70% of participants classified as obese and relatively high mean BMI values in both pain chronicity groups. This restricted distribution of adiposity may have reduced the ability to detect associations or between-group differences in body composition. Therefore, the absence of statistically significant associations should not be interpreted as evidence that adiposity is clinically unimportant in chronic LBP. Within the characteristics and range of body composition represented in this cohort, psychological and functional factors showed stronger associations with HRQoL than the evaluated body-composition parameters.

Given the cross-sectional design, the direction of these relationships cannot be determined. Chronic pain and disability may reduce habitual physical activity, potentially contributing to increased adiposity and reduced muscle function. Conversely, obesity and visceral adiposity may contribute to reduced physical capacity and greater mechanical or metabolic burden, potentially contributing to persistent pain and associated pain-related processes, including catastrophizing and central sensitization. These relationships may also be bidirectional and mutually reinforcing over time. Accordingly, the present findings cannot determine whether body composition represents a cause, consequence, mediator, or correlate of chronic pain burden.

Nevertheless, these findings do not diminish the broader clinical importance of weight management, physical activity, and preservation of muscle function as components of comprehensive rehabilitation [60,61,62,63,64]. The interpretation of the present body-composition findings should also consider the absence of data on habitual physical activity, previous musculoskeletal events, and actual pain duration, all of which may be related to both body composition and chronic pain outcomes.

### 4.3. Clinical Implications

The present findings support a comprehensive biopsychosocial assessment of patients with chronic LBP. In addition to conventional clinical and functional evaluation, patient-reported measures such as the CSI-9, PCS, and RMDQ may provide complementary information regarding central sensitization, pain-related cognitions, and functional disability. Their use in rehabilitation settings may help identify patients with a greater burden of physical and mental HRQoL impairment and support more individualized treatment planning.

The observed associations of central sensitization and pain catastrophizing with both physical and mental HRQoL also support the integration of psychologically informed approaches within multidisciplinary rehabilitation. Interventions addressing pain-related beliefs and behaviors, including pain neuroscience education, cognitive–behavioral approaches, graded exposure, and self-management education, may complement conventional exercise-based rehabilitation. At the same time, the absence of statistically significant associations between the evaluated body composition parameters and HRQoL should not diminish the importance of weight management, regular physical activity, and resistance exercise for their broader cardiometabolic and musculoskeletal benefits.

Overall, these findings support an individualized biopsychosocial rehabilitation framework in which psychological pain-related factors are considered alongside physical, functional, and body composition characteristics. Such an integrated assessment may help inform patient stratification and personalized rehabilitation planning. Prospective interventional studies are needed to determine whether targeting these factors can improve HRQoL.

### 4.4. Study Limitations

Several limitations should be considered when interpreting the present findings. First, the cross-sectional design precludes conclusions regarding causal or temporal relationships among central sensitization, pain catastrophizing, body composition, and HRQoL. Accordingly, it cannot be determined whether these factors precede poorer HRQoL or reflect consequences of long-standing chronic pain. Bidirectional relationships are also plausible, whereby persistent pain and disability may reduce physical activity and contribute to changes in adiposity and muscle function, while obesity, reduced physical capacity, and metabolic or mechanical burden may contribute to persistent pain and associated pain-related processes, including catastrophizing and central sensitization. Therefore, the observed associations should not be interpreted as establishing causal direction or the relative causal importance of psychological, functional, or body-composition factors.

Second, this was a single-center study of patients referred for rehabilitation, which may limit the generalizability of the findings to other clinical settings or to individuals with less severe chronic LBP. The study population also consisted predominantly of older adults and women, potentially limiting the applicability of the findings to younger populations and men. The use of the ICDS represents an additional limitation. Although the ICDS was used to characterize the degree of pain chronicity in the present cohort, its psychometric properties and cross-cultural validity remain insufficiently documented outside Italian-speaking populations, and the instrument has not been specifically validated in a Romanian population. Therefore, findings based on ICDS scores and subgroup categories should be interpreted cautiously, particularly with regard to their generalizability to other clinical and cultural settings.

Body composition was assessed using BIA, which, although practical and non-invasive, is not considered a reference method for body-composition assessment and provides indirect estimates that may be influenced by hydration status and other pre-measurement conditions. Although participants were instructed to avoid vigorous physical activity, alcohol consumption, and large meals before assessment, residual variation in hydration status could have affected impedance-derived estimates. Reference methods such as dual-energy X-ray absorptiometry (DXA) or magnetic resonance imaging (MRI) would provide more precise characterization of adiposity and skeletal muscle mass and should be considered in future studies. The SMI used in the present study was derived from whole-body skeletal muscle mass rather than appendicular skeletal muscle mass. Because muscle strength and physical performance were not specifically assessed for sarcopenia diagnosis, participants could not be classified as having sarcopenia according to contemporary consensus criteria. Furthermore, the high prevalence of obesity and relatively restricted distribution of adiposity in this cohort may have limited the ability to detect associations between body-composition parameters and HRQoL or differences according to pain chronicity status. The exploratory analysis of potential effect modification by diabetes should also be interpreted cautiously, as the study was not specifically designed or powered to detect interaction effects, and the limited number of participants with diabetes (*n* = 25) may have reduced the statistical power to identify such relationships. In addition, several potentially important confounders were not assessed, including habitual physical activity, actual pain duration beyond the minimum inclusion criterion of 12 weeks, previous musculoskeletal events or conditions (such as fragility fractures, major lower-extremity fractures, arthroplasty, or previous spinal surgery), fear-avoidance beliefs, anxiety, depression, sleep quality, medication use, and socioeconomic characteristics. These factors may influence body composition, functional capacity, pain perception, and HRQoL and may therefore have contributed to residual confounding in the present analyses.

Although the principal associations remained evident after additional adjustment for age and sex, residual confounding cannot be excluded because the multivariable models included a limited set of covariates, and other potentially relevant clinical, metabolic, and lifestyle factors were not comprehensively incorporated into the adjusted analyses. Therefore, the observed associations should be interpreted as adjusted for the variables included in the respective models rather than as demonstrating independence from all potential confounders.

Finally, although the multivariable models explained a substantial proportion of the variability in physical and mental HRQoL, other biological, psychological, and social factors not included in the analyses may also be relevant. The findings should therefore be interpreted within the broader biopsychosocial complexity of chronic LBP rather than as evidence that any individual factor determines HRQoL.

Despite these limitations, an important strength of the study is the simultaneous assessment of central sensitization, pain catastrophizing, functional disability, pain chronicity, body composition, and HRQoL within the same rehabilitation cohort. The integration of psychometric, functional, and body composition measures with multivariable analyses allowed these factors to be examined concurrently in relation to physical and mental HRQoL.

### 4.5. Future Directions

Future research should prioritize prospective longitudinal studies to clarify the temporal relationships among central sensitization, pain catastrophizing, body composition, and HRQoL in patients with chronic LBP. Larger multicenter studies involving more diverse demographic and clinical populations are also needed to confirm the present findings and improve their generalizability. Incorporating additional factors, including physical activity, psychological comorbidities, sleep quality, inflammatory biomarkers, and more precise body composition assessment methods, may provide a more comprehensive understanding of the biological, psychological, and functional factors associated with HRQoL.

Randomized controlled trials are needed to determine whether interventions targeting central sensitization and pain catastrophizing, particularly when integrated with exercise-based rehabilitation, result in clinically meaningful improvements in HRQoL. Future studies could also develop and validate multidimensional prognostic models combining psychological, functional, and body composition parameters to improve patient stratification and support more individualized rehabilitation strategies.

## 5. Conclusions

In this rehabilitation cohort, psychological pain-related factors, particularly central sensitization and pain catastrophizing, showed stronger statistical associations with HRQoL than the evaluated body-composition parameters. Although excess adiposity was highly prevalent, body fat percentage, visceral fat level, and SMI showed weak or non-significant associations with HRQoL and were not significantly associated with either the physical or mental components of the SF-36 in the respective adjusted models. These findings should be interpreted in the context of the obesity-enriched cohort, in which restricted variability in adiposity may have limited the ability to detect body-composition associations.

Patients with advanced pain chronicity exhibited significantly greater central sensitization, pain catastrophizing, functional disability, and poorer HRQoL, while the evaluated body-composition parameters remained largely unchanged. However, these findings should not be interpreted as indicating greater causal importance of psychological or pain-processing factors relative to body-composition characteristics. The cross-sectional design does not establish temporal or causal relationships, and the observed relationships may be bidirectional, with chronic pain, physical activity, disability, body composition, catastrophizing, and central sensitization potentially influencing one another over time.

The present results support the potential utility of assessing central sensitization and pain catastrophizing as part of the comprehensive evaluation of patients with chronic LBP. Incorporating validated patient-reported outcome measures into clinical practice may help identify patients with greater HRQoL impairment and inform patient stratification and individualized biopsychosocial rehabilitation planning. However, the present findings do not establish that interventions specifically targeting central sensitization or pain catastrophizing will improve HRQoL. Future longitudinal and interventional studies are warranted to determine whether interventions targeting these factors lead to clinically meaningful and sustained improvements in patient outcomes.

## Figures and Tables

**Table 1:** Baseline demographic, anthropometric, clinical, and laboratory characteristics of the study population.

Characteristic	Overall (*N* = 97)
**Demographic characteristics**	
Age, years	66.6 ± 9.2
Age range, years	45–84
Age, median (IQR), years	68 (61–73)
Female sex, *n* (%)	72 (74.2)
Rural residence, *n* (%)	57 (58.8)
**Anthropometric characteristics**	
Height, cm	160.8 ± 9.6
Weight, kg	84.4 ± 18.9
BMI, kg/m^2^	33.1 ± 6.1
Normal weight, *n* (%)	8 (8.2)
Overweight, *n* (%)	21 (21.6)
Obesity, *n* (%)	68 (70.1)
**Clinical characteristics**	
Vertebrogenic pain, *n* (%)	65 (67.0)
Discogenic pain, *n* (%)	12 (12.4)
Mixed pain, *n* (%)	19 (19.6)
Lumbar spinal stenosis, *n* (%)	1 (1.0)
CCI	3.0 (2.0–5.0)
Diabetes, *n* (%)	25 (25.8)
**Laboratory parameters**	
ESR, mm/h	18.0 (11.0–28.0)
Total cholesterol, mg/dL	194.2 ± 43.8
Triglycerides, mg/dL	116.0 (89.0–136.0)

Note: Data are presented as mean ± SD, median (IQR), range, or number (%), as appropriate.

**Table 2:** Psychometric characteristics, disability, pain chronicity, and HRQoL of the study population.

Characteristic	Value
**Psychometric assessment**	
CSI-9 (0–36)	18.9 ± 7.4
PCS total score (0–52)	22.8 ± 13.8
Rumination	8.3 ± 4.2
Magnification	5.3 ± 3.8
Helplessness	9.3 ± 6.7
**Disability and pain chronicity**	
RMDQ	10.1 ± 6.2
ICDS (0–14)	8.0 (5.0–11.0)
Higher degree of pain chronicity (ICDS ≥ 8), *n* (%)	56 (57.7)
**HRQoL (SF-36)**	
PF	45.4 ± 25.1
RP	18.0 ± 30.4
BP	34.6 ± 18.7
GH	43.9 ± 17.6
VT	50.7 ± 15.3
SF	54.9 ± 21.4
RE	27.1 ± 37.4
MH	65.6 ± 13.9
SF-36 PCS	35.5 ± 17.0
SF-36 MCS	49.6 ± 15.2

Note: Data are presented as mean ± SD, median (IQR), or number (%), as appropriate. Higher scores on the SF-36 indicate better HRQoL, whereas higher scores on the CSI-9, PCS, RMDQ, and ICDS indicate greater symptom burden, disability, or pain chronicity.

**Table 3:** Body composition parameters assessed by whole-body BIA.

Characteristic	Value
Body fat, %	31.8 ± 10.9
Visceral fat level	11.9 ± 5.1
Whole-body skeletal muscle mass, kg	30.4 ± 5.7
Whole-body SMI, kg/m^2^	8.5 ± 1.3
Body water, %	38.1 ± 7.9
Basal metabolic rate, kcal/day	1610.1 ± 301.0
Metabolic age, years	66.5 ± 13.6
Bone mass, kg	2.7 ± 0.5

Note: Data are presented as mean ± SD. SMI was calculated as whole-body skeletal muscle mass (kg) divided by height squared (m^2^) and does not represent ASMI. Sarcopenia status was not determined because appendicular skeletal muscle mass, muscle strength, and physical performance were not specifically assessed for sarcopenia diagnosis.

**Table 4:** Comparison of patients according to pain chronicity status.

Characteristic	ICDS < 8 (*n* = 41)	ICDS ≥ 8 (*n* = 56)	*p* Value
**Demographic characteristics**			
Age, years	65.4 ± 8.8	67.5 ± 9.4	0.28
Female sex, *n* (%)	29 (70.7)	43 (76.8)	0.49
BMI, kg/m^2^	32.4 ± 5.9	33.6 ± 6.3	0.36
CCI	3 (2–4)	3 (2–5)	0.61
**Psychometric assessment**			
CSI-9	14.8 ± 5.4	21.8 ± 6.5	<0.001
PCS total score	15.9 ± 10.7	27.8 ± 13.1	<0.001
Rumination	5.9 ± 3.4	10.0 ± 4.1	<0.001
Magnification	3.5 ± 2.8	6.7 ± 3.7	<0.001
Helplessness	6.4 ± 4.9	11.1 ± 6.8	<0.001
**Disability and HRQoL**			
Roland–Morris	6.5 ± 4.5	12.7 ± 5.8	<0.001
SF-36 PCS	45.2 ± 14.3	28.3 ± 15.2	<0.001
SF-36 MCS	56.7 ± 12.4	44.2 ± 14.3	<0.001
**Body composition**			
Body fat, %	30.9 ± 10.4	32.5 ± 11.2	0.47
Visceral fat level	11.3 ± 4.8	12.4 ± 5.4	0.34
Whole-body SMI, kg/m^2^	8.6 ± 1.2	8.4 ± 1.3	0.51

**Table 5:** Spearman rank correlation sensitivity analysis for selected psychometric, pain-chronicity, and HRQoL variables.

Variables	Spearman ρ	*p* Value
ICDS vs. CSI-9	0.440	<0.001
ICDS vs. PCS total score	0.430	<0.001
ICDS vs. RMDQ	0.370	<0.001
ICDS vs. SF-36 PCS	−0.480	<0.001
ICDS vs. SF-36 MCS	−0.370	<0.001

**Table 6:** Primary multivariable linear regression analyses of factors associated with physical and mental HRQoL.

Independent Variable	SF-36 PCS Standardized β	*p* Value	SF-36 MCS Standardized β	*p* Value	VIF
CSI-9	−0.39	<0.001	−0.34	0.002	2.4
PCS total score	−0.28	0.004	−0.29	0.005	2.0
RMDQ	−0.24	0.011	−0.15	0.100	1.8
Body fat (%)	−0.07	0.290	−0.05	0.480	1.6
SMI	0.05	0.430	0.06	0.400	1.5

Note: Model performance: SF-36 PCS model, adjusted R^2^ = 0.61, *p* < 0.001; SF-36 MCS model, adjusted R^2^ = 0.49, *p* < 0.001. VIF values < 5 were considered indicative of the absence of problematic multicollinearity.

**Table 7:** Sensitivity analyses of factors associated with HRQoL after additional adjustment for age and sex.

Variable	SF-36 PCS B (95% CI)	SF-36 PCS β	*p* Value	SF-36 MCS B (95% CI)	SF-36 MCS β	*p* Value	VIF
CSI-9	−0.58 (−0.86 to −0.30)	−0.36	<0.001	−0.49 (−0.81 to −0.17)	−0.31	0.004	2.45
Pain catastrophizing (PCS total score)	−0.21 (−0.36 to −0.06)	−0.26	0.007	−0.23 (−0.40 to −0.06)	−0.27	0.009	2.12
RMDQ	−0.38 (−0.69 to −0.07)	−0.22	0.018	−0.23 (−0.55 to 0.09)	−0.13	0.160	1.86
Body fat (%)	−0.07 (−0.25 to 0.11)	−0.05	0.430	−0.05 (−0.22 to 0.12)	−0.04	0.550	1.92
SMI	0.18 (−0.37 to 0.73)	0.04	0.520	0.21 (−0.36 to 0.78)	0.05	0.470	1.78
Age	−0.09 (−0.25 to 0.07)	−0.08	0.250	−0.07 (−0.23 to 0.09)	−0.06	0.380	1.64
Sex (female vs. male)	−1.20 (−3.84 to 1.44)	−0.06	0.370	−1.39 (−4.10 to 1.32)	−0.07	0.310	1.71

Note: B represents the unstandardized regression coefficient; 95% CI, 95% confidence interval; β, standardized regression coefficient. SF-36 PCS model: adjusted R^2^ = 0.60, *p* < 0.001; SF-36 MCS model: adjusted R^2^ = 0.48, *p* < 0.001. VIF values ranged from 1.64 to 2.45, indicating no problematic multicollinearity. Sex was coded as 0 = male and 1 = female.

**Table 8:** Sensitivity analyses of factors associated with HRQoL incorporating visceral fat level as the adiposity measure.

Variable	SF-36 PCS B (95% CI)	SF-36 PCS β	*p* Value	SF-36 MCS B (95% CI)	SF-36 MCS β	*p* Value	VIF
CSI-9	−0.56 (−0.84 to −0.28)	−0.35	<0.001	−0.48 (−0.80 to −0.16)	−0.30	0.004	2.38
Pain catastrophizing (PCS total score)	−0.21 (−0.36 to −0.06)	−0.26	0.007	−0.22 (−0.39 to −0.05)	−0.26	0.011	2.08
RMDQ	−0.37 (−0.68 to −0.06)	−0.21	0.020	−0.22 (−0.54 to 0.10)	−0.12	0.174	1.83
Visceral fat level	−0.12 (−0.39 to 0.15)	−0.06	0.380	−0.10 (−0.39 to 0.19)	−0.05	0.490	1.74
SMI	0.17 (−0.38 to 0.72)	0.04	0.540	0.20 (−0.37 to 0.77)	0.05	0.490	1.72
Age	−0.08 (−0.24 to 0.08)	−0.07	0.310	−0.07 (−0.23 to 0.09)	−0.06	0.390	1.68
Sex (female vs. male)	−1.15 (−3.80 to 1.50)	−0.06	0.390	−1.32 (−4.04 to 1.40)	−0.07	0.340	1.69

Note: B represents the unstandardized regression coefficient; 95% CI, 95% confidence interval; β, standardized regression coefficient. SF-36 PCS model: adjusted R^2^ = 0.59, *p* < 0.001; SF-36 MCS model: adjusted R^2^ = 0.47, *p* < 0.001. All VIF values were <5. Sex was coded as 0 = male and 1 = female.

**Table 9:** Exploratory analysis of the potential modifying effect of diabetes on the association between visceral adiposity and HRQoL.

Term	SF-36 PCS B (95% CI)	*p*	SF-36 MCS B (95% CI)	*p*
Visceral fat	−0.10 (−0.38 to 0.18)	0.48	−0.08 (−0.37 to 0.21)	0.59
Diabetes	−1.80 (−5.10 to 1.50)	0.28	−2.10 (−5.60 to 1.40)	0.24
Visceral fat × diabetes	−0.07 (−0.34 to 0.20)	0.61	−0.09 (−0.37 to 0.19)	0.52

Note: B represents the unstandardized regression coefficient; 95% CI, 95% confidence interval. Models were adjusted for CSI-9 score, pain catastrophizing, RMDQ score, SMI, age, and sex. Diabetes was coded as 0 = no and 1 = yes.

## Data Availability

The data presented in this study are available from the corresponding author upon reasonable request. The data are not publicly available due to privacy and ethical restrictions.

## Abbreviations

The following abbreviations are used in this manuscript:

LBP	Low Back Pain
HRQoL	Health-Related Quality of Life
CSI-9	Central Sensitization Inventory-9
PCS	Pain Catastrophizing Scale
RMDQ	Roland–Morris Disability Questionnaire
ICDS	Italian Chronic Pain Classification
SF-36	36-Item Short Form Health Survey
SF-36 PCS	SF-36 Physical Component Summary
SF-36 MCS	SF-36 Mental Component Summary
BIA	Bioelectrical Impedance Analysis
BMI	Body Mass Index
CCI	Charlson Comorbidity Index
ESR	Erythrocyte Sedimentation Rate
PF	Physical Functioning
RP	Role Limitations due to Physical Health
BP	Bodily Pain
GH	General Health
VT	Vitality
SF	Social Functioning
RE	Role Limitations due to Emotional Problems
MH	Mental Health
SMI	Skeletal Muscle Index
SD	Standard Deviation
IQR	Interquartile Range
DXA	Dual-Energy X-ray Absorptiometry
MRI	Magnetic Resonance Imaging
VIF	Variance Inflation Factor
ASMI	Appendicular Skeletal Muscle Mass Index
